# Multiple Levels of Immunological Memory and Their Association with Vaccination

**DOI:** 10.3390/vaccines9020174

**Published:** 2021-02-19

**Authors:** Zsófia Bugya, József Prechl, Tibor Szénási, Éva Nemes, Attila Bácsi, Gábor Koncz

**Affiliations:** 1 Department of Immunology, Faculty of Medicine, University of Debrecen, H-4032 Debrecen, Hungary; bugyazsofia5@gmail.com (Z.B.); szenatiborr@gmail.com (T.S.); etele@med.unideb.hu (A.B.); 2 R&D Laboratory, Diagnosticum Zrt, H-1047 Budapest, Hungary; jprechl@gmail.com; 3 Clinical Center, Department of Pediatrics, University of Debrecen, H-4032 Debrecen, Hungary; enemes@med.unideb.hu

**Keywords:** immunological memory, vaccination, B cell, T cell, elderly, newborn

## Abstract

Immunological memory is divided into many levels to counteract the provocations of diverse and ever-changing infections. Fast functions of effector memory and the superposition of both quantitatively and qualitatively plastic anticipatory memory responses together form the walls of protection against pathogens. Here we provide an overview of the role of different B and T cell subsets and their interplay, the parallel and independent functions of the B1, marginal zone B cells, T-independent- and T-dependent B cell responses, as well as functions of central and effector memory T cells, tissue-resident and follicular helper T cells in the memory responses. Age-related limitations in the immunological memory of these cell types in neonates and the elderly are also discussed. We review how certain aspects of immunological memory and the interactions of components can affect the efficacy of vaccines, in order to link our knowledge of immunological memory with the practical application of vaccination.

## 1. Introduction

Upon reinfection, the immune system faces a number of challenges. It needs to provide a rapid response via an adequate number of cells with high-affinity antigen receptors, and to further develop an immune arsenal against upcoming infections, even against variable pathogens. Immunological memory is divided into many levels to counteract such provocations. The innate immune system can retain epigenetic and transcriptional changes, resulting in altered immune responses on subsequent encounters [1]. This phenomenon is called *trained innate immunity* [2], which is remarkably different from conventional immunological memory (Table 1). Conventional immunological memory affects the repertoire of lymphocyte antigen receptors and defines our immune profile, as determined by our lifetime encounters with antigens. However, most of these encounters are silent and go unnoticed, triggering only the so-called natural immunity which we call *natural memory* in this review. Cells responsible for this type of immunity are usually antigen experienced persistent cells with antigen receptors recognizing common environmental antigens. While *natural memory* can provide a broad general defense, for survival, it is necessary to be prepared for pathogens that surpass this protection level. More focused protection is achieved by the generation of *effector memory* cells that are more potent in the elimination of targets, and by *anticipatory memory* cells that are more sensitive in the detection of specific dangerous targets. These goals are realized by increasing the frequency of antigen specific lymphocyte clones and by the polarization of the response via differentiation of lymphocytes. In the following sections, we briefly summarize the main aspects of trained immunity, but shall focus on the cells that contribute to the lymphocyte-based conventional memory, presenting the role of different B and T cell subsets and emphasizing the differences between the primary and secondary responses.

Active immunization seeks to activate immunological memory without causing pathological damage. Optimally, vaccines induce multiple levels of immunological memory. First, to provide rapid antibody response to directly block access to the pathogen and then if the pathogen breaks through the first line of defense, to activate anticipating memory T and B cells to protect against disease development. While we summarize the overlapping and collaborative steps of immune memory, we shall highlight the role of these different cell types and events in vaccination, taking into consideration the age-dependence of the immunological memory generation as well.

## 2. Trained Immunity

The term trained immunity refers to the phenomenon that occurs when, after exposure to a pathogen, the cells of the innate immune system (monocytes, macrophages, NK cells, etc.) are capable of mounting a heightened, nonspecific response against a broad range of pathogens in the case of secondary exposure [3,4]. In the initiation of trained immune phenotype, stimulation by various pattern recognition receptors results in genome wide epigenetic changes in these cells. This process leads to an altered transcriptional profile and the rewiring of the cellular metabolism [4], shifting from oxidative phosphorylation towards glycolytic processes (Warburg effect) [3]. Compared to their unexposed counterparts, these primed cells are more effective at eliminating infections both by direct (phagocytosis) and indirect (IL-1β, IL-6, TNF-α, and other cytokine production) mechanisms [5]. The phenomena of trained immune responses were first described in monocytes, macrophages and NK cells; however, more recent studies have further expanded antigen nonspecific immune memory to other innate immune cells, such as polymorphonuclear leukocytes or innate lymphoid cells (ILCs) [6]. The epigenetic and transcriptional changes associated with trained immunity can be detected up to one year [3]. Since the half-life of leukocytes in the circulation is much shorter [7], it is hypothesized that not only mature immune cells but also bone marrow stem- and progenitor cells can undergo “training” [8].

### Consequences on Vaccination

While there is no vaccine currently that directly aims to elicit a trained immune phenotype, it seems that we have been reaping the benefits of “training” through vaccination for decades. Indeed, the first evidence for the memory like properties of the innate immune system comes from vaccine studies [3]. The Bacillus Calmette-Guerin (BCG) or oral polio vaccines, which contain live attenuated microbes, given at young age, have been shown to reduce infant mortality and morbidity to a greater extent than would be expected from vaccination with these pathogens alone [9,10]. Harnessing trained immunity may lead to the development of a new generation of prophylactic vaccines. Modulation of innate immune cells can enhance both specific and nonspecific immune responses. These responses may provide protection against infections with bystander pathogens during a functional period of trained immunity [11].

## 3. Natural Memory

Specialized lymphocyte subsets, B1 cells and gamma-delta T cells (T_γδ_), are responsible for natural memory. These cells appear early in ontogenesis, are capable of self-renewal, and are promiscuous in reactivity. These poly-specific cells preferentially recognize self-antigens and conserved microbial antigens and typically elicit a rapid or even an immediate response against a limited set of antigens. B1 cells secrete cross-reactive antibodies produced even prior to the first appearance of the pathogen (Table 2).

Of the B lymphocytes B1b cells are the source of T-independent (TI) memory B lymphocytes in mice, producing low affinity antibodies in a constant, but inducible way. Nonmutated B1b lymphocytes may provide long lasting TI memory responses against the polysaccharide capsule of *Streptococcus pneumoniae* [12] and to *Borrelia hermsii* [13]. B1 cells can differentiate into both memory B cells [12] and plasma cells [14] and these memory responses can provide protection upon the transfer of these cells to immunodeficient hosts [13].

A special population of the T lymphocytes, T_γδ_, is programmed for effector functions very early in the thymus [15]. There are two major groups of T_γδ,_ which produce IFNγ and IL-17A, and contribute to the protection against intracellular and extracellular pathogens, respectively. The skin, the gut and the reproductive tract are seeded by these cells early on in the developing fetus, thereby providing the first line of defense. Human T_γδ_ cells have limited specificity and generally use only two variable gene segments for their delta chains and are classified into Vδ1 and Vδ2 cells. Vδ1 T cells are found primarily at epithelial sites, while Vδ2 T cells are dominant among circulating Tγδ cells [16]. Importantly, all naive Vδ2 cells disappear from the blood by 1 year of age and the non-naive cells show potent effector functions allowing rapid reactions to a limited group of recognized antigens [17]. The restricted specificity and oligoclonality in the intestinal Vδ repertoire also indicates that these cells represent memory in the intestinal tract against various recurrent ligands [18].

### Consequences on Vaccination

Cells responsible for maintaining a natural, baseline protectivity establish threshold immunity against all available antigens. These cells possess self-renewing capability and are in a continuous minimal activations state, which also allows them to bypass conventional lymphocyte differentiation pathways. Thus, vaccination ideally induces the generation of memory cells that exceed this threshold immunity with regard to antigen elimination efficiency. Importantly, owing to the unique antigen-receptor signaling mechanisms in these cells, natural memory remains functional, even when effector memory cells appear and eliminate that particular antigen [19,20].

## 4. Overview of B Cell-Mediated Immune Response

Antibodies produced by plasma cells provide B-cell-mediated immune protection. First, short-lived plasma cells are formed upon contact with antigens and secrete low-affinity immunoglobulin M (IgM) antibodies for a few days, followed by apoptosis [21,22]. Not only can these cells play an important role against primary infections, but they also prevent reinfections in a short period of time. Different levels of B cell-mediated immunity are involved in the memory response. Soluble antibodies, produced by long-lived plasma cells (LLPC) as *effector memory* cells, ensure the first lines of immune defense. Immunoglobulin A (IgA) secreted into mucosa can prevent the pathogen from entering the human body, while the appearance of other immunoglobulin isotypes (IgG, IgM) of antibodies in tissues or in the circulation can prevent pathogens from entering human cells, or mark invaders for destruction. LLPC producing immunoglobulin E (IgE) responses have evolved for protection against helminth parasites infections, but IgE is also a potent mediator of allergic diseases [23]. Optimally high affinity, specific antibodies are secreted by LLPC even before the pathogen enters. This type of protection usually prevents infections (called sterilizing immunity) unless an extreme dose of the pathogen invades the host that goes beyond the effects of protective antibodies.

Memory B cells also contribute to B cell-mediated defense in reinfections, as *anticipatory memory* cells. In contrast to LLPC, memory B cells express BCR on their cell surface and are therefore able to detect pathogens. Once encountered with antigens, these cells undergo clonal proliferation and mostly differentiate into short-lived plasma cells. This memory B cell-mediated response does not provide immediate protection, but is still significantly faster than the response of naive B cells. Memory B cells support plasma cell-mediated protection on at least three levels after reinfection. (1) The ability of these cells to proliferate and differentiate provides unlimited replenishment for the terminally differentiated, nondividing LLPC pool. (2) Memory B cells can undergo affinity maturation and this allows a further increase of the quality of the response. (3) These cells also provide flexibility in the B cell memory response, since low-affinity memory B cells may also be able to recognize altered pathogens. Both memory B cells and LLPC cells have a long lifespan, even without reinfection. The survival of the two cell populations is an independent process, LLPC may persist after elimination of peripheral B cells and are not sequentially replaced from the memory B cell pool [24]. The adoptive transfer of any of these populations generates protection in the host upon reinfection [25].

As most pathogens carry both protein and nonprotein epitopes, memory cells and LLPCs can be produced in three parallel ways: in a T-independent manner, and in a T-dependent manner either outside B-cell follicles or in the GCs of B-cell follicles. Theoretically, the B-cell response consists of successive stages: B1 cell-mediated innate immunity, marginal zone (MZ) B cells, extrafollicular and intrafollicular B cells, sequentially from lower affinity to higher affinity and faster to slower response. While these events are anatomically and functionally different, all of these cell types may contribute to both the production of antibodies and the formation of memory B cells. All of these effects can be considered during vaccination, it is presumably rational to find a balance between the induction of shorter and less focused responses versus long-lasting and sharply defined responses, taking into consideration the properties of the pathogen, of the host and also the epidemiology.

## 5. MZ B Cells and T-Independent B Cell Memory 

B cells are able to recognize various antigen substances, but T cell activation is mostly restricted to peptide recognition. The TI B cellular response is mostly against saccharide or lipid antigens and is characterized by the rapid emergence of low-affinity antibodies. Optimal B cell memory response relies on the supportive signals of cognate helper T cells. Consequently, B cell memory develops primarily against protein antigens, but various evidence suggests the existence of effective memory responses even after the T-independent activation of B cells [26,27]. The formation of both TI long lived memory cells [28] and plasma cells have been published [14]. Hapten-specific memory and plasma cells survived for up to 100 days for both saccharides [28,29] and LPS [30] antigens, even in T cell-deficient, and germinal center (GC)-deficient mice [30]. Polysaccharide antigens of encapsulated bacteria are also used successfully in human vaccination. PNEUMOVAX® 23, the 23-valent anti-pneumococcal vaccine, provides protection in adults and children more than 2 years of age [31,32] and the protection against *Haemophilus influenzae type b* is also effective due to antibodies against polysaccharide-type capsules [33]. 

MZ B cells are complementary actors with B-1b cells in the humoral response to TI Ag. Vaccine-induced TI B-cell memory is likely to be mostly derived from MZ B-cells. The expansion of MZ B cells has been shown following immunization with pneumococcal polysaccharide vaccine [34] and the lack of circulating MZ B cells makes humans highly susceptible to infection with *H. influenzae type b* [35]. Without T cell help, somatic hypermutation cannot take place, consequently, the amount of responding cells grows, instead of their affinity increasing in B1 and MZ cell-mediated memory response [27].

Conventional B2 cells may also contribute to TI Ab responses, as B cell specificity develops in a random procedure. However, the function and survival of these classical B cells may be deterministically more dependent on T cell help. It is known that T-cell-derived signals, such as CD40 activation or IL-21 production are generally required for class switching, somatic hypermutation (SHM), and enhancement of B-cell proliferation. Therefore, the TI B cell response generally does not produce high affinity antibodies, is less intense, and is likely to be transient [26,28,36].

An additional limitation of the T-independent response is that these reactions are more age-dependent. Development of MZ B cells does not occur under 2 years of age. Polysaccharide-based vaccines have had limited use in newborns and because the response of MZ B cells is primarily spleen-specific it was also missing in spleen-deficient individuals [37], until the introduction of conjugate vaccines that circumvent T cell incompetence against saccharide antigens.

### Consequences on Vaccination

Although most T-independent plasma cells are short-lived, protein-free antigens can also induce memory B cells and long-lived plasma cell formation such as it was evidenced by the usage of *S. pneumoniae* [38], *H. influenzae type b*, or *Neisseria meningitidis* polysaccharide vaccines [39].

In the absence of protein epitopes or in immunocompromised individuals, this response may be of significant importance. However, after reinfection with complex antigens (which express both protein and nonprotein epitopes), high-affinity T-dependent memory B cells can outcompete T-independent memory B cells. Consequently, T-independent B cells generally have no or only a marginal role in the secondary response. In addition, negative regulatory feedback of antigen-specific immunoglobulins keeps TI memory B cells under control [28]. Under very specific conditions, when the first immunization activates only saccharide-specific B cells (such as it can be in the case of *S. pneumoniae* polysaccharide vaccines, PPSV23) the number of saccharide-specific B cells increases and thus these cells may dominate the secondary response even after immunization with complex antigens [40].

Age related structural changes in the spleen and lymph nodes [41] results in a substantial loss of marginal zone [42] also in the elderly. The decline in the function of MZ B cells [43] with age is less intense than the downregulation of T dependent response, so a polysaccharide vaccine PPSV23 is still used in the elderly over the age of 65 years [44].

## 6. T-Dependent, Extrafollicular B Cell Activation

Adaptive immunity plays a dual role in the immune response, providing protection against current infections and creating memory cells to prepare the immune system for upcoming infections. Accordingly, T-dependent B cell activation occurs by two main mechanisms. The principal purpose of extrafollicular B cell activation is to generate a rapid response to eliminate the current pathogens, while intrafollicular, GC-related B cell responses primarily serve for long-term protection [45,46,47,48].

The first phase of T and B cell interaction begins at the junction of the T cell-zone and B cell follicles, where the activated B cells present the specific antigen to the T cells already activated by dendritic cells (DCs) [47]. A few hundred short-lived plasma cells (plasmablasts) are typically formed in these foci within a few days after the first encounter with the antigen [45]. Characteristically, high-affinity B cells from the available repertoire prefer extrafollicular plasmablasts differentiation [49,50] to provide protection rapidly, while the originally lower-affinity, but still antigen specific, B cells migrate to the follicles [51,52].

The interaction with T cells is also important for extrafollicular B cells, as it mediates support and induces the expression of activation-induced deaminase (AID) [53], the enzyme regulates antibody class switching and SHM. Although these processes are not completely ruled out [48], extrafollicular plasmablasts typically do not undergo SHM and affinity maturation [45,54]. In contrast, antibody class switching frequently occurs in these cells during the perifollicular proliferative phase [49,55].

Extrafollicular B cells are predominantly short-lived, their half-life is only a few days [48], however, these B cells can also induce early memory B cell [56] and LLPC differentiation [49,57]. These memory B cells develop independently of GC within a few days of the response [58]. Like all conventional memory B cells, these cells are quiescent, long-lived cells and are able to produce larger amounts of antibodies during recall than naive B cells [59]. Once differentiated, their survival is independent of T cells and the continuous presence of related antigens [45,60,61].

### Consequences on Vaccination

Since active immunization focuses only on future infections, extrafollicular reactions which aim to serve actual protection, have a limited role in vaccination. We have already mentioned in the T-independent memory section, that in the secondary response, high-affinity T-dependent memory B cells are able to outcompete lower affinity counterparts, including extrafollicular memory B cells. In general, extrafollicular memory B cells do not contribute significantly to the long term secondary antibody response [45] unless GC formation is intensely blocked. Some infections (such as *Borrelia burgdorferi*, and *Ehrlichia muris*) have been reported to elicit an intense extrafollicular response while suppressing GCs in the meantime. SARS-CoV-2 infection also blocks T cell function in the GCs and consequently reduces GC formation with the accumulation of non-germinal-center-derived activated B cells [62].

## 7. T Dependent B Cell Memory, Intrafollicular B Cell Activation

**Figure 1:**
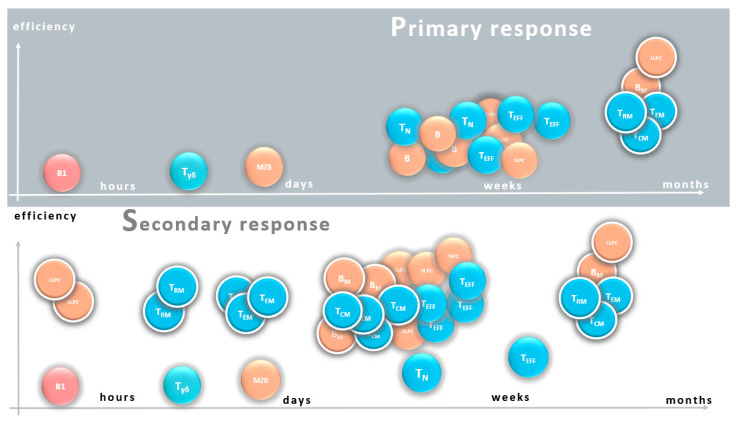
Phases of antigen specific immune response in primary and secondary response.

The follicular GC reaction is initiated by antigen-specific B cells and the infiltrating antigen-specific follicular helper T cells (T_FH_) [49,50]. B cells here undergo affinity maturation during time-consuming reactions, resulting in a drastic increase in their affinity in the following weeks (Figure 1).

In the primary response, natural immunity provides minimal protection within hours, which in turn determines the threshold of response. Marginal zone B cells (MZ) are able to react in a few days to produce low-affinity antibodies. The intense proliferation of naive T and B cells and their differentiation into effector cells, short-lived plasma cells (SLPC) and effector T cells (T_EF_), eliminates infections in about a week. Development of antigen specific long-lived cells (with white rim), memory B cells (B_M_), long-lived plasma cells (LLPC), central (T_CM_) and tissue-resident (T_RM_), and effector memory T cells (T_EM_) will be completed within weeks, and provides long-term protection.

In the secondary response, an increased number of antigen-specific cells are ready to initiate the response. High-affinity antibodies produced by LLPC induce an immediate response that may result in sterilizing immunity. Due to the optimal localization of T_RM_, an efficient T cell response develops immediately after antigen presentation. A relatively larger number of T_EM_ cells enter from the circulation at the site of infection even in a day. High numbers of B_M_ and T_CM_ begin to proliferate within a few days in secondary lymphatic organs, resulting in quick replenishment of effector cells. The efficiency of the response is increased due to the higher affinity antibodies and their optimal isotype, as well as the increased reactivity of T cells. A new generation of long-lived cells may also develop, even in larger numbers and with higher affinity antibody production.

The absolute threshold of the B cell affinity for selection is unknown, selection for antigen binding depends rather on the relative affinity of competing clones [63,64]. Antigens in the form of immune complexes are localized on the surface of the FDC in the follicles [65,66], but antigen presentation to T cells requires BCR-mediated internalization of foreign antigens, processing into peptide fragments, and binding to MHC-class molecules on the B cell surface [47,67]. B cells with relatively higher affinity may consume all antigens or at least block access to antigen-rich sites of FDCs from other B cells. B cell–B cell interactions cannot be ruled out in GC, and as a result, higher-affinity B cells can rip off the antigen from those with lower affinity. The presentation of more MHC-peptide complexes to T_FH_ cells provides a competitive advantage with stronger feedback or a higher probability of survival [63,68] and these T-cell-mediated supportive signals are dominant in the positive selection of B cells [66,69]. The BCR stimulus alone, without successful antigen presentation, rather induces GC B cell apoptosis, whereas B cells capable of presenting antigen-derived peptides to T_FH_ receive survival and proliferation signals [47]. Although T cell-derived signals determine the fate of competing clones, it should be noted that the number of antigen presented is directly proportional to the affinity of the BCR [70,71]. 

Interaction with cognate T cells activates CD40-induced signaling, which stimulates AID that controls somatic mutation and isotype switching. Selected high-affinity B cells undergo clonal proliferation while SHM occurs, resulting in different affinities of developing clones. New clones with partially different affinities may return and compete again for antigens and T cell help. While cells with higher affinity are selectively enriched in each cycle, cells with lower affinity are eliminated by apoptosis, consequently, B cell affinity increases in a stepwise fashion in each additional cycle in the GCs. At the end of the process, the affinity of GC B cells far exceeds the affinity of extrafollicularly activated B cells [47]. The number of cycles and how they are controlled is currently unknown. Positively selected B cells can differentiate in three ways after interaction with T cells at the end of each cycle. These cells can return to start a new selection cycle for further diversification or can be exported from GC as LLPC or as B memory cells. 

The B cells with the highest affinity differentiate primarily into LLPCs in the follicles, but it is still not clear what factors determine whether a B cell stays in the GC or exits as a memory cell. Class-switched memory cells do not differ from GCs B cells either in affinity or in the number of somatic mutations [50].

Relatively low affinity clones of memory B cells may also survive to provide flexibility in the immune response. As a result, the sensitivity of memory B cells against a given antigen covers a broad spectrum of affinity from low affinity clones, typically expressing IgM, to cells expressing higher affinity IgG, thereby preparing the body to respond to mutations in pathogens [72,73].

After reinfection, basically, the same process takes place as in the primary response, but with higher speed and improved quality. The antibodies produced by LLPCs not only inactivate pathogens, but also facilitate the formation of immune complexes and thus the delivery of antigens to the FDCs [74]. A large number of B memory cells have a competitive advantage over naive cells, and higher affinity clones (primarily expressing IgG) initiate short lived plasma cell differentiation for rapid protection. Lower affinity memory cells (typically with IgM expression) initiate cyclic responses in the newly formed GC, which results in sequentially higher affinity long-lived memory B cells and LLPCs after each repeated infection [75,76,77].

### Consequences on Vaccination

The majority of responding memory cells and LLPCs against T-dependent antigens are likely to be derived from GCs, and express BCR with higher affinity than naive B cells. Most vaccines use exclusively LLPC as correlates (for example toxoid induced immunization or conjugated vaccines, HPV, etc.) or LLPC together with memory B cells or memory T cells (among many others smallpox, influenza, varicella-zoster, *Bordetella pertussis*, BCG) [78,79]. If the amount, effectiveness and localization of antibodies (mostly IgG and IgA) are sufficient to prevent the colonization of pathogens, vaccines result in sterilizing immunity such as vaccines against polio, rubella, *H. influenzae type b*, *S. pneumoniae* and others [80].

It must be noted that elevated specific antibody levels do not automatically provide protection. While the neutralizing effect of antibodies is undoubtedly beneficial for vaccination, when an antibody associates with a pathogen without its functional inactivation, it can facilitate phagocytosis as an opsonin and consequently, the entry of the microbe into human cells, a phenomenon called “Antibody-Dependent Enhancement” or ADE. It has been found that antibodies and even vaccine-induced antibody responses, can have an adverse effect in the case of various infections [81], such as influenza [82] or SARS vaccinations [83].

It is important to note that the rate of pathogen spreading can be deterministic in deciding whether memory B cells can be considered as effective correlates. The protection of fast-dividing pathogens relies on LLPC, while the spread of slower pathogens gives enough time for memory B cells to proliferate and differentiate, such as in the case of hepatitis B and probably of SARS-CoV-2 viruses [84]. In the case of an extremely slow pathogen spread (such as rabies), there is sufficient time for a vaccine to elicit a primary response and in these cases, the active immune response may be protective even when given after infection [85] (called post-exposure prophylaxis).

The memory B cells not only outcompete any naive cells, but reduce the minimal antigen requirements for initiating a response [86]. These additional advantages of memory B cells over naive B cells are also exploited in booster injection used in most immunization procedures [87]. The limited number of antigens in the vaccine does not necessarily trigger the formation of LLPC during the first exposure, since the generation of these cells requires high affinity interactions, but memory B cells formed in the meantime may sensitize the secondary response [47].

Although the relationship between B cell affinity and the lifespan of memory B cells or LLPCs is not exactly known, we can assume that a response initiated with higher affinity clones results in a longer lifespan. Both the activation of the transcription factors required for the development of LLPC and the chance of achieving an optimal tissue environment (i.e., bone marrow) depend on BCR affinity [88]. If this correlation is relevant in determining longevity, then after primary infection with a lower dose of the antigen, when only available naive B cells respond, relatively short-lived cells are formed. However secondary infection activates memory cells with already higher affinity, thus creating long-lived memory and plasma cells. As the selection process to create high affinity cells in GCs lasts for weeks, antigen persistence may be deterministic in affinity maturation, highlighting the importance of the depot effect of the adjuvant. However, chronic reactions can lead to lymphocyte depletion, so determining the optimal time window may be important for the development of ideal B-cell responses [89,90].

We must consider that most antigens express a number of different epitopes. Immunization with complex antigens leads to the activation and competition of B cells of different specificities [63]. Repeated B cell responses against quickly changing pathogens such as influenza virus, result in a restriction to act only to unmodified epitopes, as B memory cells outcompete naive B cells that attack newly emerging epitopes. Accordingly, B-cell clones activated during the first infection may also dominate the response to upcoming influenza infections [91]. By sequentially limiting the recognized targets, modified pathogen strains can eventually avoid B-cell memory response, a phenomenon called original antigenic sin [91]. The emergence of new variations observed with SARS-CoV-2 raises concerns that similar processes may occur with this pathogen, especially for vaccines that focus on only one protein to induce protection. If different epitopes were chosen as dominant epitopes in different individuals, it might limit the spread of pathogens in the human population, but in the meantime might make it difficult to establish a widely effective vaccine.

## 8. Overview of T Cell-Mediated Immune Response

In addition to B cells, the other type of lymphocyte that expresses antigen receptors and contributes to memory responses is the T cell. Most T cells recognize the antigens presented by other cells. Glycolipids are represented by a class of CD1 molecules, while conventional T cells, which belong to either the CD8 or CD4 population, recognize MHCI and MHCII peptides of the major histocompatibility complex. Following activation, T cells act on host cells through direct cell–cell contact or secreted cytokines.

Antigen-specific naive T cells require antigen transport from the site of infection into the secondary lymphatic organs (SLO) and antigen presentation by activated/mature DCs. Consequently, the activation of innate immunity is a prerequisite for naive T cell activation, and T-dependent B cell activation. This initial contact between DCs and T cells depends on the expression of costimulatory molecules on stimulated DCs. Subsequently, naive T cells differentiate into effector T cells during clonal proliferation. It is important to note that costimulation is not required to activate effector T cells, so the effector phase of the response is not necessarily dependent on innate immune cells.

An important aspect of T cell activation for optimal vaccination is that intracellular pathogens must also be transported and presented in SLO, a process that is predominantly performed by DCs. Dead cells phagocytosed by DCs are the source of antigen, as direct infections of DCs are very rare. Following the phagocytosis of destroyed human cells, DCs are able to present the absorbed antigen by MHCI and MHCII molecules during so-called cross-presentation, which results in the activation of both cytotoxic and helper T cells. 

Helper T cells are classified according to their functional characteristics. TH1, TH2, TH17 cells direct the immune response mainly against intracellular pathogens, multicellular parasites, and extracellular pathogens (bacteria and fungi), respectively, with distinctive cytokine production in each class. Certain helper T cell subsets have special functions, such as follicular helper T (T_FH_) cells to interact with B cells [92] or regulatory T cells to induce immune tolerance. Effective vaccination should mimic the proper TH subtype response generated by the target pathogen. In non-live vaccines, this is primarily determined by adjuvant-mediated activation of innate immunity.

As compared to the primary immune response, T cells show quantitative and qualitative differences during these recall responses. Memory T cells will remain overrepresented in the repertoire and this quantitative difference enhances the probability of an encounter with the antigen. During the primary response, chromatin modifications render activation-inducible genes ready for transcription in memory cells [93,94]. Thus, upon the secondary response, the differentiation of the cells is accompanied by polarization and changes in homing patterns, the cells will have preferentially accumulated in tissues where they are needed and will deploy cytokines and cytotoxic measures as needed, all leading towards faster and more effective immunity.

The development of memory cells is similar in each T cell type, so unless otherwise indicated, reference is made below to both CD4 subtypes and CD8 cells. T cell memory relies on many different T memory cell types. Gamma-delta T cells and effector T memory cells provide a faster rate of memory response compared to primary immunization, while central memory T cells, following clonal proliferation are the source of newly formed effector T cells during reinfection and also provide a degree of flexibility in the T cell memory response (Figure 1).

## 9. The Effector Cells in T Cell Memory

The first line of defense provided by memory T cells is the immediate action towards the removal of target cells. This is brought about by cells that are continuously looking for targets and can instantly release cytokines or induce cell death: tissue resident memory T cells (T_RM_) (systematically reviewed [95,96]), and the somewhat later acting effector memory T cells (T_EM_).

### 9.1. Tissue Resident Memory T Cells 

T_RM_, unlike circulating memory cells, remains localized in peripheral tissues defined by the previous infections, by downregulating tissue egress receptors [97] and expressing tissue specific adhesion molecules [98]. T_RM_ cells have the same TCR repertoire as central memory T cells (T_CM_), so it is not specificity but localization that makes this cell population unique [96]. This is a heterogeneous population of cells owing to differences in localization (skin, mucosal sites, brain, and liver) and may show plasticity in their differentiation.

T_RM_ expresses effector molecules like granzyme B, TNFα and IFNγ thereby providing quick protection against invading pathogens [99]. In general, the CD4^+^ T_RM_ response has a lag-phase because secreted cytokines need time to take effect, while a CD8^+^ T_RM_ response may represent quick cytotoxic action, if antigen specific cells are present in sufficiently large numbers. Low random migration of T_RM_ cells may considerably enhance the ability of the T_RM_ cells to scan their environment [96].

T_RM_ cells can persist for long periods in the absence of the cognate antigen. The number of T_RM_ is limited in a given tissue, and T_RM_ cell populations of different specificities may also compete for niche, survival and growth factors [96]. Repetitive stimulation may provide an advantage for antigen specific clones in this competition [100]. Repeated infection can induce a local response where the in situ proliferation of T_RM_ generates a secondary T_RM_ population besides the cells differentiating from recruited cells [101,102], thus the regulation of local memory independently of central memory is possible. However, the overall limited number of T_RM_ cells may be critical, mainly against rapidly replicating pathogens in terms of direct effects. It can be hypothesized that the main function of T_RM_ is not to eliminate pathogens directly, but this accelerated antigen detection can facilitate the recruitment of circulating T_EM_ and T_CM_ cells by the induced cytokine production. Killing some infected cells may also accelerate cross-presentation by producing dead cells for DC-mediated phagocytosis, facilitating the antigen presentation of intracellular pathogens by DCs [96,103]. Parenterally administered vaccines, which dominate in current formulations, should be ideally enhanced in their ability to induce T_RM_.

### 9.2. Effector Memory T Cells

The other population of memory T cells, which circulates in the blood and is present in nonlymphoid, peripheral tissues is the T_EM_ [104]. T_EM_ cells, which are present in relatively large numbers but do not have proliferative potential, can exit into tissues (T_RM_ can also be important in determining the location of an exit) and survey peripheral tissues for a specific antigen, where they can quickly exert effector functions or return to the blood via lymphatics [99]. T_EM_ cells lack the lymph node homing receptors [105] and constitutively display effector functions such as IFNγ production and cytotoxic activity [106]. 

T_EM_ and T_RM_ are equipped with the arsenal of cytokines and cytolytic mechanisms necessary to maintain a balance between the host and pathogens. Once an invasion has been averted and the immune response has contracted, these cells continue to survey the tissues and eliminate remaining or newly encountered pathogens. Should the target breach this barrier again, destroy tissues and multiply, these cells are not able to proliferate. This is when cells anticipating such events step in.

## 10. The Anticipatory Memory Cells in T Cell Memory

### 10.1. Central Memory T Cells

The second line of defense is formed by memory T cells that can quickly divide and differentiate into powerful effector cells. These cells possess a central phenotype, localizing to and recirculating between secondary lymphoid organs. T_CM_ are comparable to naive T cells with respect to their homing pattern and proliferative burst upon antigenic stimulation [107]. However, T_CM_ are much more sensitive to activation stimuli owing to having been selected with high affinity TCR, changes in the composition and clustering of signaling molecules and epigenetic differences allowing rapid transcription of genes [108]. Accordingly, it provides replenishment for T_EM_ cells relatively quickly following reinfection.

Another type of T cell with central phenotype is the follicular helper T cell (T_FH_) (systematic reviews in [109,110]). Though these cells reside in the follicles of secondary lymphoid organs, where they provide signals to differentiating B cells, T_FH_ orchestrates GC reactions and are indispensable for TD memory B-cell responses. Like conventional memory cells, T_FH_ cells have a long lifespan and when transferred between mice in in vivo experiments, also provide memory for the recipients [109,110]. Two cooperating mechanisms provide long-term protection by these cells. First, T_FH_ cells are able to survive for up to 400 days after infection and also have self-renewing potential [111,112]. This subset is rapidly activated after reinfection and their differentiation into effector cells does not require antigen presentation by DCs, only by memory B cells [109]. Second, T_FH_ cells may have a central memory phenotype and at certain stages of development they are also present in the circulation [113,114]. Circulating memory T_FH_ cells have a less T_FH_-polarized phenotype than local memory T_FH_ cells [114]. The T helper memory response is substantially plastic, with TH subtypes repolarizing to other populations upon antigen recall [115]. Overall, T_FH_ cells maintain anticipatory immunological memory by helping the development of secondary B-cell response [112], as discussed above.

### 10.2. Memory Stem T Cells

T_S_**_CM_** are minimally differentiated, self-renewing, long-lived cells with significant reconstitution potential even after the disappearance of the antigen. These cells show phenotypic, distribution and recirculation pattern similarity to naive T cells, yet they are clonally expanded, also express several characteristic cell surface markers of memory cells and create a rapid response to antigen [116]. T_SCM_ can be differentiated directly from naive cells by omitting the effector phase [100]. T_SCM_ are generated during the immune response against various pathogens and are precursors of other memory T cells. T_SCM_ seems to have critical importance in maintaining life-long cellular immunity [117,118], thus ideally clinical vaccine formulations should be able to induce T_SCM_ along with other memory compartments, in order to induce long-lasting T-cell memory [119]. 

### 10.3. Consequences on Vaccination

Most vaccines focus on the plasma cell response and the optimal production of neutralizing antibody. Unilateral overrepresentation of plasma cells during vaccination may obscure the beneficial or critical functions of other memory cells (Table 3). The reaction of memory T cells as well as memory B cells is unable to provide immediate protection and cannot prevent infections, but these mechanisms are fast and effective enough to prevent the development of the disease, thereby protecting individuals and also protecting the population by reducing the duration of infection. T cell mediated protection at least partially correlates with vaccine efficacy in various vaccines such as in BCG, measles, varicella-zoster virus, rabies, and in many others [78,79].

Cytotoxic T cells are essential for immune protection against intracellular pathogens, and accordingly are important correlates in vaccination. For example, T-cell deficiency leads to severe and fatal disease in measles, while B-cell deficient people recover [80]. As the cytotoxic T cell response begins with cross-presentation, vaccine derived antigen must be intracellular (as live, DNA or RNA vaccine) to be engulfed and properly presented by DCs. Among others, considerable long lasting cytotoxic T cell memory has been published following SARS-Cov-2 infection [84], and at least in rhesus macaques T cells may contribute to the protection in collaboration with antibodies [120]. Vaccine antigens that are not expressed intracellularly (e.g., killed pathogens or some subunit vaccines) are unable to activate cytotoxic T cells, and these cells cannot differentiate to memory cells during such vaccinations.

The dose of the antigen delivered during immunization is critical for both cytotoxic and helper T cell activation. High antigen concentrations can result in T cell exhaustion or clonal deletion of highly reactive T cells through activation-induced cell death (AICD). In contrast, a low antigen dose primarily leads to the expansion of high-affinity CD8 T cells [121], while relatively weaker TCR signals also efficiently produce memory CD8 T cells [122]. The selection, activation, and polarization of helper T cells also depend on the intensity of immunization [123,124]. 

The role of T_RM_ in initiating a secondary response highlights the importance of finding the optimal route of vaccine administration that results in the proliferation of T_RM_ cells in tissues where an upcoming infection may start. The longevity and reactivity of T_FH_ cells directly regulate the response of memory B cells, which may thus affect the development of the immune response during reinfection, but even during booster vaccination [125]. The polarization of TH cells to different populations is mostly influenced by adjuvants, which in some cases may reduce the efficiency of the immune response, such as that reported for the acellular pertussis vaccine, where the mixed TH1/TH2-directed immune response is activated instead of the optimal TH17 [126].

Effective T cell memory is particularly important against highly variable pathogens. While epitopes available for B cells (often on the surface of the pathogen) are generally frequently changed, peptides involved in antigen presentation may be derived from more conservative structural elements. Reinfection results in all T_RM_, T_EM_ and T_CM_ activity recognizing mutation-free, conserved epitopes. These cells may also be reactive even after infection with newly emerging, antigen-shifted virus strains as was highlighted for example in influenza epidemics [127].

T cell-related reactions, in parallel with or in combination with ADE, may contribute to vaccine-associated disease enhancement (VADE). Live vaccines such as dengue, RSV vaccines, or vaccines against coronaviruses may also increase susceptibility to viral infection by a mechanism that has not yet been fully elucidated. Vaccine-induced altered cytokine profile, inflammatory damage, TH2 activation, and subsequent eosinophil infiltration can be responsible for aberrant viral pathogenesis [128,129].

## 11. Age-Dependence of Immunological Memory

**Figure 2:**
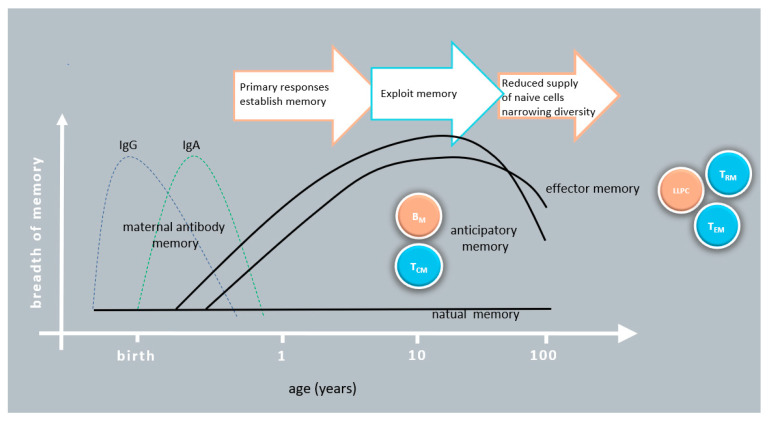
Changes of elements of memory response during life.

The development of the immune system starts already in utero, but it is after birth that exposure to the abundance of environmental antigens and danger signals initiates immunological memory formation. This cumulative phase of memory corresponds to the diversification and tuning of immune responses and goes on until early adulthood. Following decades of maintenance of immune function in general, memory efficacy and diversity start to wane, typically at the age of 65–70 years. Due to the distinct characteristics of early and late years of life, we briefly summarize the critical changes that characterize the immune memory response in neonates and in the elderly (Figure 2).

Early in life the maternal immune memory provides protection by IgG and IgA transport and transfer. Memory cell levels are very low in neonates and only reach the intensity typical of adults around the age of 10–15 years. Weaker antigen receptor and costimulatory signals in the first years of life favor the development of anticipatory rather than effector memory. Memory efficacy and diversity start to wane, typically at the age of 65–70 years. After this age, the elderly respond poorly to new infections and memory response is also limited. The restricted repertoire of antigen specific cells primarily limits protection to recurrent infections.

## 12. Neonatal Immunity

Immune maturation continues into adolescence, but the most significant changes occur in the first years of life. The immune system of newborns is typically tolerogenic, which influences memory cell formation and vaccination at several points. Basically, the number of lymphocytes at birth is high [130], but with unique characteristics in functionality in neonates. As there is no pathogenicity in the uterus, memory cell levels are very low and remain limited for years [131].

Maternal immunological experience is transferred to a newborn by two means. IgG transfer from the mother to the fetus occurs during pregnancy across the placenta, while IgA is transferred to the neonatal gastrointestinal tract by breastfeeding. These antibodies not only protect but also direct the developing immune system towards target molecules, and are therefore expected to have a strong impact on the establishment of immunological memory of the offspring [132].

Humoral immunity is impaired in neonates. Although B-cell levels are high at birth [130] and the repertoire is quite diverse, most of the cells are still immature B cells (approximately 95% of the B cells) that only slowly transform into peripheral mature B cells [133] and do not reach the adult level till childhood (5–10 years). Isotype switched and memory B cells are very rare in newborns and at 1 year of age, infant IgG levels are 70%, while IgA levels are even lower, only 30% of the adults [133]. The T-independent B cell response is severely limited, among other things, because the peripheral zone of the spleen is not fully developed by the age of 2 years and the level of CD21 receptor (complement receptor 2) on B cells is reduced [133,134]. Germinal center reactions, which are critical for the long-term memory response, are highly compromised. Weaker B cell receptor-mediated signaling in naive B cells, incomplete help provided by follicular dendritic cells, and limited expansion of T_FH_ cells together result in suboptimal B cell activation in GCs [135,136].

The number of memory B cells increases slowly with age and reaches adult levels only in children aged 10–15 years [137]. Thus, higher sensitivity of memory cells, i.e., higher affinity of memory B cells or lower dependence on costimulation of memory T cells, cannot be exploited in neonate vaccination. Weaker BCR and TCR dependent signaling results in the dominance of memory B cells formation versus plasma cells and insufficient survival factors available LLPC further exacerbate this phenomenon [135].

At birth, the number of T cells is high and increases steadily for years, and then begins to decline until it reaches adult levels in older childhood [130]. Although the T cell number is high, their function is inadequate. The functional activity of neonatal APCs is impaired, and lower expression of costimulatory molecules complicates the activation of naive T cells, rather enhances the possibility of an anergic T cell response [138]. In addition, functional exhaustion is also characteristic of neonatal effector T cells [139]. In neonates CD8 + T cells are less responsive and require a greater degree of antigen specific stimuli [140]. In contrast, the activity of regulatory T cells is upregulated. These cells are present in large amounts in human cord blood and are highly tolerogenic in their function [141,142,143]. This overall decrease in antigen-specific response complicates the formation of both memory B cells and memory T cells. Not only does the strength of the T cell response decrease, but the balance of the response is also strongly skewed toward Th2 polarization [144]. Overall reduced IFN and IL-12 production leads to decreased TH1 differentiation, highlighting the importance of using adjuvants capable of regulating T cell polarization during vaccination [131].

### Consequences on Vaccination

Newborns are highly vulnerable to infectious diseases. It is therefore important to develop the most effective and safest vaccines possible for them. The highly tolerogenic immune system, decreased strength of antigen receptor signaling, lack of pre-existing memory cells, severely impaired GC function, TH imbalance, and the presence of the maternal neutralizing antibody all counteract successful vaccination. The need for early intervention and the suboptimal circumstances for vaccination require thoughtful strategies [145].

Nonetheless, some vaccines are already suitable for newborns, and some, moreover, are the most effective in this age. An example is the BCG vaccine, which is one of the first and most widely used vaccines [146]. BCG does not need to contain any exogenous adjuvants because the live attenuated strain of *Mycobacterium bovis* induces immune responses via many PRRs [147]. BCG induces a strong TH1 response demonstrating that decreased TH1 capacity in neonates is not absolute [148]. There is growing evidence that BCG perhaps induces maturation of dendritic cells, affects TH cytokine balance and influences the heterologous trained immunity [10]. Many studies suggest that early immunization with BCG may protect against, inter alia, leukemia, allergies, and childhood diabetes and that BCG elevates cytokine and antibody responses to other vaccine antigens (HBV and OPV) in neonates [149,150,151]. The BCG vaccine is not only an example of a vaccine that can function under the tolerogenic conditions, typically present in newborns, but also of vaccines that can direct the maturation of the immune system. 

A special challenge in the vaccination of newborns is the prevention of the transmission of pathogens from mother to infant [152]. The HBV vaccine induces at least as much antibody production in neonates as in adults and gives a lifetime protection after a minimum of three doses. The HBV vaccine represents that subunit vaccines with adjuvant (aluminum salts) can effectively function in newborns while oral polio vaccine demonstrates active intestinal immune response and the potential success of mucosal vaccination [145]. Extending maternal immunization, which has already been successful with various vaccines among others tetanus-diphtheria-pertussis, influenza, hepatitis B, could be a promising strategy to solve the problem of early infections [153].

The special characteristic of newborn immunity has to be considered in vaccination, for example in T-cell independent B-cell response to polysaccharide antigens. The delay in the development of the peripheral zone of the spleen, decreased CD21 receptor levels on B cells, lower complement activity [154], unbalanced cytokine profile [155], and increased susceptibility to tolerance induction in neonates may also play a role in suboptimal reactions to encapsulated bacteria [154,155]. These mechanisms can be circumvented with conjugate vaccines that switch the reactions to T-dependent response.

Experience with these existing vaccines suggests that in spite of the restricted memory response neonates are able to develop efficient immune response, especially to live attenuated vaccines, but these immune responses depend on several factors, such as maternal Ab at the time of administration, breastfeeding, timing of postpartum vaccination, and geographical location [133].

## 13. Immunity in the Elderly

The adaptive immune system undergoes numerous alterations with age and the last stage of life is characterized by relevant changes in the immune response. When the first exposure to an antigen occurs at an older age, a less effective immune response is induced. As a result, a weaker memory response is generated to new antigens and the success of vaccination is reduced in elderly. Long-lived memory cells (both T and B cells) do not just persist, but even protect the elderly, however, the diversity of the repertoire of memory cells narrows and their responsiveness also deteriorates [122,156,157,158,159,160]. 

In the bone marrow, the production of B cells decreases as internal changes appear that preclude commitment to the lymphoid line, IL-7 survival factor production decreases, the proliferation rate regresses and apoptotic capacity increases [161]. The number of naive B cells in the periphery is also declining in older adults [162]. In addition to the decrease in number, the function of aging B cells is also impaired. Antigen-induced plasma cell differentiation and antigen-specific antibody production are reduced [163,164], although total levels of serum antibodies are rather increased in the elderly [165]. Internal defects in germinal centers, including decreased AID production studied in mice, have been identified, resulting in impaired mechanisms of both class-switching recombination CSR and SHM [166,167]. All of these processes limit the way memory B cells and LLPC are formed. As these changes have a lesser effect on TI B cells, the decrease in TI responses with age is less severe than that of TD responses [44].

B memory cells accumulate in the periphery and the population of B cells in the periphery is saturated with memory B-cells over time. However, even in the presence of increased autoantibody levels, these memory B cells have limited repertoire diversity and exhibit paralyzed functions. A part of memory B cells differentiate to exhausted memory cells with poor ability to replicate and likely prone to apoptosis [168]. Reduced function of T_FH_ cells, decreased production of AID and impaired immune-complex retention of FDC compromise memory B cell functions [41,122] and consequently limits optimal plasma cell formation [165]. Further exacerbating the problem, the imperfect bone marrow environment is less conducive to LLPC survival [169], accordingly, BM stores fewer plasma cells at old age [163].

While the number of circulating T cells remains almost the same throughout life, there are decisive changes in their composition and reactivity. Due to the involution of the thymus, the development of T cells becomes independent of the thymus and a general decrease in the production and diversity of naive T cells occurs [170]. The functionality of naive T cells was also impaired, including weaker TCR and costimulatory signals, reduced proliferative capacity and decreased IL-2 production [171]. Three mechanisms compensate for the decrease in naive T cell production: (1) increased antigen-independent clonal expansion of naive T cells [172]; (2) more naive T cells (both CD4 + and CD8 +) differentiate into “virtual” memory cells [173,174], which are cells with a longer lifespan but reduced antigen reactivity; (3) memory T cells are activated from time to time by recurrent viral infections (average of 8-12 recurrent pathogens). Intermittent activation and proliferation of pathogen-specific memory T cells impairs the balance of naive and memory T cells and can account for 50% of all CD4 T cells turning memory T cells [175,176]. All of these mechanisms result in a decrease in the diversity of T cells in the elderly, even a hundred-fold decrease in the diversity of CD4 T cells has been observed after the age of 70 years [177]. Although the number of memory T cells increases with age, the diversity and reactivity of these cells also decrease, limiting the possibility of an effective response to some well-known antigens. It further limits the maintenance of T cell memory intensity that both primary and memory T cell responses generate short-lived effector T cells rather than memory precursor cells [122]. In addition to cytokine production, the cytokine balance also changes with age. Th1 responses decrease, shifting toward Th2 cytokine predominance [178]. In summary, the reactivity and plasticity of naive and memory T cells are reduced, limiting the possibility of developing a stronger immune response to an upcoming infection.

### Consequences on Vaccination

Due to the aging of the immune system, the elderly are more vulnerable to infections, resulting in higher mortality and morbidity by pathogens. Changes in memory response also result in moderate vaccine efficacy in the elderly. For example knowing that long-lived memory T and B cells can provide effective protection against influenza infection that recurs after a long time, following influenza vaccination seroconversion (a fourfold increase in antibody titer) and protective hemagglutination inhibitor antibody titers is successfully produced in only 10–30% of the elderly, compared with 50–75% of the younger [179]. Even when antibody levels rise, it has been observed that elevated levels do not persist for even a year [180]. Crippled T cell response also manifested for example in suboptimal Th1 cells response in the lung [181]. 

Changes not only in the intensity but also in the composition of the immune system may require new vaccination strategies, for example to avoid *S. pneumoniae* infection [181]. However, pneumococcal conjugate vaccine (PCV13) stimulates T cells, via conjugating the pneumococcus glycans to CRM197 (nontoxic mutant of diphtheria toxin) and accordingly this vaccine is used for people under 65 years of age [182], but for adults ≥65 years of age the PPV23 is also recommended because the effectiveness of the T-independent response decreases less with age [44].

Due to age-related immune system defects, the control of pathogen reactivation is also weakened, such as a latent varicella zoster infection can lead to herpes zoster disease. Limited susceptibility to vaccines makes it even more difficult to control such diseases at this age. Live attenuated virus vaccination against Varicella zoster virus, which is quite effective at age 60 (51%), is almost ineffective in older populations [183]. Studies showed that the immune responses to the vaccine waned in 6-8 years [184]. In summary, vaccines need to be improved to protect this highly vulnerable population through regular booster vaccinations, finding optimal doses and routes of administration or by innovative adjuvants and inventing new technologies.

## 14. Conclusions

Social alterations in humanity increase the global risk of pandemics, which demand more effective vaccination. As the scope of the article highlights, the memory response relies on a wide variety of cell populations, with their different localizations, affinities, reaction times, and flexibility. Although neutralizing antibody production is the only way to generate sterilizing immunity, other cells and other mechanisms of immunological memory can/should be considered during vaccination. The variety and variability of pathogens requires the plasticity of the responses used against them. In addition, the heterogeneity of the human population, in terms of age, immune status, and comorbidities, may necessitate the development of several vaccines against the same pathogen. These challenges require a more accurate understanding of the complex processes of immunological memory, all of which can make targeted approaches in vaccination.

## Figures and Tables

**Table 1:** Comparison of innate, natural and conventional immune memory.

Name	Memory	Cells	Mechanism	Antigen Specificity	Duration	Targets
Trained innate immunity	Innate	Mo, MF, ILC, NK	Epigenetic	No/limited	Transient	Conserved/personal
Natural immunity	Natural adaptive	B1, T_γδ_	Epigenetic and genetic	Limited	Lasting	Conserved
Immunological memory	Adaptive	B cells, T cells	Epigenetic and genetic	Huge	Lasting	Personal

**Table 2:** Comparison of naive and natural, anticipatory and effector memory response.

Characteristics	Naive	Natural Memory	Anticipatory Memory	Effector Memory
B cell types	B2	B1	B_M_	LLPC
T cell types	T_N_	T_γδ_	T_CM_, T_SCM_	T_EM_, T_RM_
Activation state	Resting	Self-renewing	Resting	Active
Function	Replenishment, seeding new memory	Natural gatekeeper	Circulating surveillance	Terminally differentiated effector
Response type	Primary response	Maintenance	Secondary response	Maintenance
Proliferative capacity	High upon activation	Constant	High upon activation	No/limited
Time of protection	Slow	Immediate/quick	Medium	Immediate/quick
Efficiency	Medium	Low	Medium to high	High
Flexibility of repertoire	High	Limited	Medium	No/minimal

**Table 3:** Strengths and weaknesses of different vaccine types in memory response in addition to B cell-related memory.

Vaccine Type	Strength in Memory Response	Weakness in Memory Response
Attenuated	Multiple antigens CTL response Optimal helper T cell response, Parallel PRR activations	
Inactivated	Multiple antigens Adjuvant determined helper T cell response Parallel PRR activations	Lack of CTL response Possibility of nonoptimal TH response May induce weaker response, may need booster
Subunit	Adjuvant determined helper T cell response	Lack of CTL response Adjuvant dependent Possibility of nonoptimal TH response Possibility of antigen sin May induce weaker response, may need booster
Conjugated	Adjuvant determined helper T cell response	Lack of CTL response Adjuvant dependent Possibility of nonoptimal TH response Possibility of antigen sin May induce weaker response, may need booster
Toxoid		Lack of CTL response (irrelevant) Adjuvant dependent Possibility of nonoptimal TH response (irrelevant) May induce weaker response, may need booster
VLP	CTL response Optimal helper T cell response Parallel PRR activations	Possibility of antigen sin The carrier-specific reaction may result in a weaker reaction
RNA	CTL response Helper T cell response PRR activations	Possibility of antigen sin May induce weaker response, may need booster
DNA	CTL response Helper T cell response	Possibility of antigen sin May induce weaker response, may need booster

## Data Availability

Not applicable.

## References

[1] Dominguez-Andres J., Netea M.G. (2020). The specifics of innate immune memory. Science.

[2] Boraschi D., Italiani P. (2018). Innate Immune Memory: Time for Adopting a Correct Terminology. Front. Immunol..

[3] Netea M.G., Joosten L.A., Latz E., Mills K.H., Natoli G., Stunnenberg H.G., O’Neill L.A., Xavier R.J. (2016). Trained immunity: A program of innate immune memory in health and disease. Science.

[4] Riksen N.P., Netea M.G. (2018). Be aware, innate immune cells remember. Aging.

[5] Kleinnijenhuis J., Quintin J., Preijers F., Benn C.S., Joosten L.A., Jacobs C., van Loenhout J., Xavier R.J., Aaby P., van der Meer J.W. (2014). Long-lasting effects of BCG vaccination on both heterologous Th1/Th17 responses and innate trained immunity. J. Innate Immun..

[6] Netea M.G., Latz E., Mills K.H., O’Neill L.A. (2015). Innate immune memory: A paradigm shift in understanding host defense. Nat. Immunol..

[7] Patel A.A., Zhang Y., Fullerton J.N., Boelen L., Rongvaux A., Maini A.A., Bigley V., Flavell R.A., Gilroy D.W., Asquith B. (2017). The fate and lifespan of human monocyte subsets in steady state and systemic inflammation. J. Exp. Med..

[8] Mitroulis I., Ruppova K., Wang B., Chen L.S., Grzybek M., Grinenko T., Eugster A., Troullinaki M., Palladini A., Kourtzelis I. (2018). Modulation of Myelopoiesis Progenitors Is an Integral Component of Trained Immunity. Cell.

[9] Garly M.L., Martins C.L., Bale C., Balde M.A., Hedegaard K.L., Gustafson P., Lisse I.M., Whittle H.C., Aaby P. (2003). BCG scar and positive tuberculin reaction associated with reduced child mortality in West Africa. A non-specific beneficial effect of BCG?. Vaccine.

[10] Pollard A.J., Finn A., Curtis N. (2017). Non-specific effects of vaccines: Plausible and potentially important, but implications uncertain. Arch. Dis. Child..

[11] Sanchez-Ramon S., Conejero L., Netea M.G., Sancho D., Palomares O., Subiza J.L. (2018). Trained Immunity-Based Vaccines: A New Paradigm for the Development of Broad-Spectrum Anti-infectious Formulations. Front. Immunol..

[12] Haas K.M., Poe J.C., Steeber D.A., Tedder T.F. (2005). B-1a and B-1b cells exhibit distinct developmental requirements and have unique functional roles in innate and adaptive immunity to *S. pneumoniae*. Immunity.

[13] Alugupalli K.R., Leong J.M., Woodland R.T., Muramatsu M., Honjo T., Gerstein R.M. (2004). B1b lymphocytes confer T cell-independent long-lasting immunity. Immunity.

[14] Taillardet M., Haffar G., Mondiere P., Asensio M.J., Gheit H., Burdin N., Defrance T., Genestier L. (2009). The thymus-independent immunity conferred by a pneumococcal polysaccharide is mediated by long-lived plasma cells. Blood.

[15] Parker M.E., Ciofani M. (2020). Regulation of gammadelta T Cell Effector Diversification in the Thymus. Front. Immunol..

[16] Deusch K., Luling F., Reich K., Classen M., Wagner H., Pfeffer K. (1991). A major fraction of human intraepithelial lymphocytes simultaneously expresses the gamma/delta T cell receptor, the CD8 accessory molecule and preferentially uses the V delta 1 gene segment. Eur. J. Immunol..

[17] De Rosa S.C., Andrus J.P., Perfetto S.P., Mantovani J.J., Herzenberg L.A., Herzenberg L.A., Roederer M. (2004). Ontogeny of gamma delta T cells in humans. J. Immunol..

[18] Holtmeier W., Witthoft T., Hennemann A., Winter H.S., Kagnoff M.F. (1997). The TCR-delta repertoire in human intestine undergoes characteristic changes during fetal to adult development. J. Immunol..

[19] Ochsenbein A.F., Fehr T., Lutz C., Suter M., Brombacher F., Hengartner H., Zinkernagel R.M. (1999). Control of early viral and bacterial distribution and disease by natural antibodies. Science.

[20] Jayasekera J.P., Moseman E.A., Carroll M.C. (2007). Natural antibody and complement mediate neutralization of influenza virus in the absence of prior immunity. J. Virol..

[21] Smith K.G., Hewitson T.D., Nossal G.J., Tarlinton D.M. (1996). The phenotype and fate of the antibody-forming cells of the splenic foci. Eur. J. Immunol..

[22] Auner H.W., Beham-Schmid C., Dillon N., Sabbattini P. (2010). The life span of short-lived plasma cells is partly determined by a block on activation of apoptotic caspases acting in combination with endoplasmic reticulum stress. Blood.

[23] Saunders S.P., Ma E.G.M., Aranda C.J., Curotto de Lafaille M.A. (2019). Non-classical B Cell Memory of Allergic IgE Responses. Front. Immunol..

[24] Hammarlund E., Thomas A., Amanna I.J., Holden L.A., Slayden O.D., Park B., Gao L., Slifka M.K. (2017). Plasma cell survival in the absence of B cell memory. Nat. Commun..

[25] Slifka M.K., Antia R., Whitmire J.K., Ahmed R. (1998). Humoral immunity due to long-lived plasma cells. Immunity.

[26] Bortnick A., Allman D. (2013). What is and what should always have been: Long-lived plasma cells induced by T cell-independent antigens. J. Immunol..

[27] Defrance T., Taillardet M., Genestier L. (2011). T cell-independent B cell memory. Curr. Opin. Immunol..

[28] Obukhanych T.V., Nussenzweig M.C. (2006). T-independent type II immune responses generate memory B cells. J. Exp. Med..

[29] Hsu M.C., Toellner K.M., Vinuesa C.G., Maclennan I.C. (2006). B cell clones that sustain long-term plasmablast growth in T-independent extrafollicular antibody responses. Proc. Natl. Acad. Sci. USA.

[30] Bortnick A., Chernova I., Quinn W.J., Mugnier M., Cancro M.P., Allman D. (2012). Long-lived bone marrow plasma cells are induced early in response to T cell-independent or T cell-dependent antigens. J. Immunol..

[31] O’Brien K.L., Hochman M., Goldblatt D. (2007). Combined schedules of pneumococcal conjugate and polysaccharide vaccines: Is hyporesponsiveness an issue?. Lancet Infect. Dis..

[32] Daniels C.C., Rogers P.D., Shelton C.M. (2016). A Review of Pneumococcal Vaccines: Current Polysaccharide Vaccine Recommendations and Future Protein Antigens. J. Pediatr. Pharmacol. Ther..

[33] Kayhty H., Peltola H., Karanko V., Makela P.H. (1983). The protective level of serum antibodies to the capsular polysaccharide of Haemophilus influenzae type b. J. Infect. Dis..

[34] Weller S., Braun M.C., Tan B.K., Rosenwald A., Cordier C., Conley M.E., Plebani A., Kumararatne D.S., Bonnet D., Tournilhac O. (2004). Human blood IgM “memory” B cells are circulating splenic marginal zone B cells harboring a prediversified immunoglobulin repertoire. Blood.

[35] Zouali M., Richard Y. (2011). Marginal zone B-cells, a gatekeeper of innate immunity. Front. Immunol..

[36] Kaminski D.A., Stavnezer J. (2006). Enhanced IgA class switching in marginal zone and B1 B cells relative to follicular/B2 B cells. J. Immunol..

[37] Breukels M.A., Zandvoort A., van Den Dobbelsteen G.P., van Den Muijsenberg A., Lodewijk M.E., Beurret M., Klok P.A., Timens W., Rijkers G.T. (2001). Pneumococcal conjugate vaccines overcome splenic dependency of antibody response to pneumococcal polysaccharides. Infect. Immun..

[38] Moberley S.A., Holden J., Tatham D.P., Andrews R.M. (2008). Vaccines for preventing pneumococcal infection in adults. Cochrane Database Syst. Rev..

[39] Patel M., Lee C.K. (2001). Polysaccharide vaccines for preventing serogroup A meningococcal meningitis. Cochrane Database Syst. Rev..

[40] Papadatou I., Tzovara I., Licciardi P.V. (2019). The Role of Serotype-Specific Immunological Memory in Pneumococcal Vaccination: Current Knowledge and Future Prospects. Vaccines.

[41] Turner V.M., Mabbott N.A. (2017). Influence of ageing on the microarchitecture of the spleen and lymph nodes. Biogerontology.

[42] Cortegano I., Rodriguez M., Martin I., Prado M.C., Ruiz C., Hortiguela R., Alia M., Vilar M., Mira H., Cano E. (2017). Altered marginal zone and innate-like B cells in aged senescence-accelerated SAMP8 mice with defective IgG1 responses. Cell Death Dis..

[43] Turner V.M., Mabbott N.A. (2017). Ageing adversely affects the migration and function of marginal zone B cells. Immunology.

[44] Shi Y., Yamazaki T., Okubo Y., Uehara Y., Sugane K., Agematsu K. (2005). Regulation of aged humoral immune defense against pneumococcal bacteria by IgM memory B cell. J. Immunol..

[45] Takemori T., Kaji T., Takahashi Y., Shimoda M., Rajewsky K. (2014). Generation of memory B cells inside and outside germinal centers. Eur. J. Immunol..

[46] Nutt S.L., Hodgkin P.D., Tarlinton D.M., Corcoran L.M. (2015). The generation of antibody-secreting plasma cells. Nat. Rev. Immunol..

[47] McHeyzer-Williams M., Okitsu S., Wang N., McHeyzer-Williams L. (2011). Molecular programming of B cell memory. Nat. Rev. Immunol..

[48] Elsner R.A., Shlomchik M.J. (2020). Germinal Center and Extrafollicular B Cell Responses in Vaccination, Immunity, and Autoimmunity. Immunity.

[49] Chan T.D., Gatto D., Wood K., Camidge T., Basten A., Brink R. (2009). Antigen affinity controls rapid T-dependent antibody production by driving the expansion rather than the differentiation or extrafollicular migration of early plasmablasts. J. Immunol..

[50] O’Connor B.P., Vogel L.A., Zhang W., Loo W., Shnider D., Lind E.F., Ratliff M., Noelle R.J., Erickson L.D. (2006). Imprinting the fate of antigen-reactive B cells through the affinity of the B cell receptor. J. Immunol..

[51] Paus D., Phan T.G., Chan T.D., Gardam S., Basten A., Brink R. (2006). Antigen recognition strength regulates the choice between extrafollicular plasma cell and germinal center B cell differentiation. J. Exp. Med..

[52] Carrasco Y.R., Batista F.D. (2007). B cells acquire particulate antigen in a macrophage-rich area at the boundary between the follicle and the subcapsular sinus of the lymph node. Immunity.

[53] Stuber E., Strober W. (1996). The T cell-B cell interaction via OX40-OX40L is necessary for the T cell-dependent humoral immune response. J. Exp. Med..

[54] MacLennan I.C., Toellner K.M., Cunningham A.F., Serre K., Sze D.M., Zuniga E., Cook M.C., Vinuesa C.G. (2003). Extrafollicular antibody responses. Immunol. Rev..

[55] Pape K.A., Kouskoff V., Nemazee D., Tang H.L., Cyster J.G., Tze L.E., Hippen K.L., Behrens T.W., Jenkins M.K. (2003). Visualization of the genesis and fate of isotype-switched B cells during a primary immune response. J. Exp. Med..

[56] Toyama H., Okada S., Hatano M., Takahashi Y., Takeda N., Ichii H., Takemori T., Kuroda Y., Tokuhisa T. (2002). Memory B cells without somatic hypermutation are generated from Bcl6-deficient B cells. Immunity.

[57] Malkiel S., Barlev A.N., Atisha-Fregoso Y., Suurmond J., Diamond B. (2018). Plasma Cell Differentiation Pathways in Systemic Lupus Erythematosus. Front. Immunol..

[58] Kaji T., Ishige A., Hikida M., Taka J., Hijikata A., Kubo M., Nagashima T., Takahashi Y., Kurosaki T., Okada M. (2012). Distinct cellular pathways select germline-encoded and somatically mutated antibodies into immunological memory. J. Exp. Med..

[59] Inamine A., Takahashi Y., Baba N., Miyake K., Tokuhisa T., Takemori T., Abe R. (2005). Two waves of memory B-cell generation in the primary immune response. Int. Immunol..

[60] Vieira P., Rajewsky K. (1990). Persistence of memory B cells in mice deprived of T cell help. Int. Immunol..

[61] Maruyama M., Lam K.P., Rajewsky K. (2000). Memory B-cell persistence is independent of persisting immunizing antigen. Nature.

[62] Kaneko N., Kuo H.H., Boucau J., Farmer J.R., Allard-Chamard H., Mahajan V.S., Piechocka-Trocha A., Lefteri K., Osborn M., Bals J. (2020). Loss of Bcl-6-Expressing T Follicular Helper Cells and Germinal Centers in COVID-19. Cell.

[63] Finney J., Yeh C.H., Kelsoe G., Kuraoka M. (2018). Germinal center responses to complex antigens. Immunol. Rev..

[64] Dal Porto J.M., Haberman A.M., Kelsoe G., Shlomchik M.J. (2002). Very low affinity B cells form germinal centers, become memory B cells, and participate in secondary immune responses when higher affinity competition is reduced. J. Exp. Med..

[65] Schwickert T.A., Lindquist R.L., Shakhar G., Livshits G., Skokos D., Kosco-Vilbois M.H., Dustin M.L., Nussenzweig M.C. (2007). In vivo imaging of germinal centres reveals a dynamic open structure. Nature.

[66] Allen C.D., Okada T., Tang H.L., Cyster J.G. (2007). Imaging of germinal center selection events during affinity maturation. Science.

[67] Batista F.D., Harwood N.E. (2009). The who, how and where of antigen presentation to B cells. Nat. Rev. Immunol..

[68] Good-Jacobson K.L., Szumilas C.G., Chen L., Sharpe A.H., Tomayko M.M., Shlomchik M.J. (2010). PD-1 regulates germinal center B cell survival and the formation and affinity of long-lived plasma cells. Nat. Immunol..

[69] Victora G.D., Schwickert T.A., Fooksman D.R., Kamphorst A.O., Meyer-Hermann M., Dustin M.L., Nussenzweig M.C. (2010). Germinal center dynamics revealed by multiphoton microscopy with a photoactivatable fluorescent reporter. Cell.

[70] Turner J.S., Ke F., Grigorova I.L. (2018). B Cell Receptor Crosslinking Augments Germinal Center B Cell Selection when T Cell Help Is Limiting. Cell Rep..

[71] Akkaya M., Kwak K., Pierce S.K. (2020). B cell memory: Building two walls of protection against pathogens. Nat. Rev. Immunol..

[72] Inoue T., Moran I., Shinnakasu R., Phan T.G., Kurosaki T. (2018). Generation of memory B cells and their reactivation. Immunol. Rev..

[73] Pape K.A., Maul R.W., Dileepan T., Paustian A.S., Gearhart P.J., Jenkins M.K. (2018). Naive B Cells with High-Avidity Germline-Encoded Antigen Receptors Produce Persistent IgM(+) and Transient IgG(+) Memory B Cells. Immunity.

[74] Garg A.K., Desikan R., Dixit N.M. (2019). Preferential Presentation of High-Affinity Immune Complexes in Germinal Centers Can Explain How Passive Immunization Improves the Humoral Response. Cell Rep..

[75] Kurosaki T., Kometani K., Ise W. (2015). Memory B cells. Nat. Rev. Immunol..

[76] Pape K.A., Taylor J.J., Maul R.W., Gearhart P.J., Jenkins M.K. (2011). Different B cell populations mediate early and late memory during an endogenous immune response. Science.

[77] Dogan I., Bertocci B., Vilmont V., Delbos F., Megret J., Storck S., Reynaud C.A., Weill J.C. (2009). Multiple layers of B cell memory with different effector functions. Nat. Immunol..

[78] Plotkin S.A. (2010). Correlates of protection induced by vaccination. Clin. Vaccine Immunol..

[79] Plotkin S.A. (2020). Updates on immunologic correlates of vaccine-induced protection. Vaccine.

[80] Plotkin S.A. (2008). Vaccines: Correlates of vaccine-induced immunity. Clin. Infect. Dis..

[81] Taylor A., Foo S.S., Bruzzone R., Dinh L.V., King N.J., Mahalingam S. (2015). Fc receptors in antibody-dependent enhancement of viral infections. Immunol. Rev..

[82] Winarski K.L., Tang J., Klenow L., Lee J., Coyle E.M., Manischewitz J., Turner H.L., Takeda K., Ward A.B., Golding H. (2019). Antibody-dependent enhancement of influenza disease promoted by increase in hemagglutinin stem flexibility and virus fusion kinetics. Proc. Natl. Acad. Sci. USA.

[83] Arvin A.M., Fink K., Schmid M.A., Cathcart A., Spreafico R., Havenar-Daughton C., Lanzavecchia A., Corti D., Virgin H.W. (2020). A perspective on potential antibody-dependent enhancement of SARS-CoV-2. Nature.

[84] Dan J.M., Mateus J., Kato Y., Hastie K.M., Yu E.D., Faliti C.E., Grifoni A., Ramirez S.I., Haupt S., Frazier A. (2021). Immunological memory to SARS-CoV-2 assessed for up to 8 months after infection. Science.

[85] Pollard A.J., Bijker E.M. (2020). A guide to vaccinology: From basic principles to new developments. Nat. Rev. Immunol..

[86] Phan T.G., Paus D., Chan T.D., Turner M.L., Nutt S.L., Basten A., Brink R. (2006). High affinity germinal center B cells are actively selected into the plasma cell compartment. J. Exp. Med..

[87] Blanchard-Rohner G., Pulickal A.S., Jol-van der Zijde C.M., Snape M.D., Pollard A.J. (2009). Appearance of peripheral blood plasma cells and memory B cells in a primary and secondary immune response in humans. Blood.

[88] Gonzalez-Garcia I., Rodriguez-Bayona B., Mora-Lopez F., Campos-Caro A., Brieva J.A. (2008). Increased survival is a selective feature of human circulating antigen-induced plasma cells synthesizing high-affinity antibodies. Blood.

[89] Castiglione F., Mantile F., De Berardinis P., Prisco A. (2012). How the interval between prime and boost injection affects the immune response in a computational model of the immune system. Comput. Math. Methods Med..

[90] Tam H.H., Melo M.B., Kang M., Pelet J.M., Ruda V.M., Foley M.H., Hu J.K., Kumari S., Crampton J., Baldeon A.D. (2016). Sustained antigen availability during germinal center initiation enhances antibody responses to vaccination. Proc. Natl. Acad. Sci. USA.

[91] Henry C., Palm A.E., Krammer F., Wilson P.C. (2018). From Original Antigenic Sin to the Universal Influenza Virus Vaccine. Trends Immunol..

[92] Tangye S.G., Ma C.S., Brink R., Deenick E.K. (2013). The good, the bad and the ugly—TFH cells in human health and disease. Nat. Rev. Immunol..

[93] Barski A., Cuddapah S., Kartashov A.V., Liu C., Imamichi H., Yang W., Peng W., Lane H.C., Zhao K. (2017). Rapid Recall Ability of Memory T cells is Encoded in their Epigenome. Sci. Rep..

[94] Chen Y., Zander R., Khatun A., Schauder D.M., Cui W. (2018). Transcriptional and Epigenetic Regulation of Effector and Memory CD8 T Cell Differentiation. Front. Immunol..

[95] Muruganandah V., Sathkumara H.D., Navarro S., Kupz A. (2018). A Systematic Review: The Role of Resident Memory T Cells in Infectious Diseases and Their Relevance for Vaccine Development. Front. Immunol..

[96] Mueller S.N., Mackay L.K. (2016). Tissue-resident memory T cells: Local specialists in immune defence. Nat. Rev. Immunol..

[97] Skon C.N., Lee J.Y., Anderson K.G., Masopust D., Hogquist K.A., Jameson S.C. (2013). Transcriptional downregulation of S1pr1 is required for the establishment of resident memory CD8+ T cells. Nat. Immunol..

[98] Mackay L.K., Rahimpour A., Ma J.Z., Collins N., Stock A.T., Hafon M.L., Vega-Ramos J., Lauzurica P., Mueller S.N., Stefanovic T. (2013). The developmental pathway for CD103(+)CD8+ tissue-resident memory T cells of skin. Nat. Immunol..

[99] Schenkel J.M., Masopust D. (2014). Tissue-resident memory T cells. Immunity.

[100] Jameson S.C., Masopust D. (2018). Understanding Subset Diversity in T Cell Memory. Immunity.

[101] Park S.L., Zaid A., Hor J.L., Christo S.N., Prier J.E., Davies B., Alexandre Y.O., Gregory J.L., Russell T.A., Gebhardt T. (2018). Local proliferation maintains a stable pool of tissue-resident memory T cells after antiviral recall responses. Nat. Immunol..

[102] Beura L.K., Mitchell J.S., Thompson E.A., Schenkel J.M., Mohammed J., Wijeyesinghe S., Fonseca R., Burbach B.J., Hickman H.D., Vezys V. (2018). Intravital mucosal imaging of CD8(+) resident memory T cells shows tissue-autonomous recall responses that amplify secondary memory. Nat. Immunol..

[103] Schenkel J.M., Fraser K.A., Beura L.K., Pauken K.E., Vezys V., Masopust D. (2014). T cell memory. Resident memory CD8 T cells trigger protective innate and adaptive immune responses. Science.

[104] Sallusto F., Lenig D., Forster R., Lipp M., Lanzavecchia A. (1999). Two subsets of memory T lymphocytes with distinct homing potentials and effector functions. Nature.

[105] Masopust D., Vezys V., Marzo A.L., Lefrancois L. (2001). Preferential localization of effector memory cells in nonlymphoid tissue. Science.

[106] Bouneaud C., Garcia Z., Kourilsky P., Pannetier C. (2005). Lineage relationships, homeostasis, and recall capacities of central- and effector-memory CD8 T cells in vivo. J. Exp. Med..

[107] Sallusto F., Geginat J., Lanzavecchia A. (2004). Central memory and effector memory T cell subsets: Function, generation, and maintenance. Annu. Rev. Immunol..

[108] Youngblood B., Hale J.S., Kissick H.T., Ahn E., Xu X., Wieland A., Araki K., West E.E., Ghoneim H.E., Fan Y. (2017). Effector CD8 T cells dedifferentiate into long-lived memory cells. Nature.

[109] Hale J.S., Ahmed R. (2015). Memory T follicular helper CD4 T cells. Front. Immunol..

[110] Crotty S. (2014). T follicular helper cell differentiation, function, and roles in disease. Immunity.

[111] Luthje K., Kallies A., Shimohakamada Y., Belz G.T., Light A., Tarlinton D.M., Nutt S.L. (2012). The development and fate of follicular helper T cells defined by an IL-21 reporter mouse. Nat. Immunol..

[112] Kunzli M., Schreiner D., Pereboom T.C., Swarnalekha N., Litzler L.C., Lotscher J., Ertuna Y.I., Roux J., Geier F., Jakob R.P. (2020). Long-lived T follicular helper cells retain plasticity and help sustain humoral immunity. Sci. Immunol..

[113] Song W., Craft J. (2019). T follicular helper cell heterogeneity: Time, space, and function. Immunol. Rev..

[114] Asrir A., Aloulou M., Gador M., Perals C., Fazilleau N. (2017). Interconnected subsets of memory follicular helper T cells have different effector functions. Nat. Commun..

[115] Lu K.T., Kanno Y., Cannons J.L., Handon R., Bible P., Elkahloun A.G., Anderson S.M., Wei L., Sun H., O’Shea J.J. (2011). Functional and epigenetic studies reveal multistep differentiation and plasticity of in vitro-generated and in vivo-derived follicular T helper cells. Immunity.

[116] Gattinoni L., Speiser D.E., Lichterfeld M., Bonini C. (2017). T memory stem cells in health and disease. Nat. Med..

[117] Gattinoni L., Lugli E., Ji Y., Pos Z., Paulos C.M., Quigley M.F., Almeida J.R., Gostick E., Yu Z., Carpenito C. (2011). A human memory T cell subset with stem cell-like properties. Nat. Med..

[118] Lugli E., Dominguez M.H., Gattinoni L., Chattopadhyay P.K., Bolton D.L., Song K., Klatt N.R., Brenchley J.M., Vaccari M., Gostick E. (2013). Superior T memory stem cell persistence supports long-lived T cell memory. J. Clin. Investig..

[119] Fuertes Marraco S.A., Soneson C., Delorenzi M., Speiser D.E. (2015). Genome-wide RNA profiling of long-lasting stem cell-like memory CD8 T cells induced by Yellow Fever vaccination in humans. Genom. Data.

[120] McMahan K., Yu J., Mercado N.B., Loos C., Tostanoski L.H., Chandrashekar A., Liu J., Peter L., Atyeo C., Zhu A. (2020). Correlates of protection against SARS-CoV-2 in rhesus macaques. Nature.

[121] Billeskov R., Beikzadeh B., Berzofsky J.A. (2019). The effect of antigen dose on T cell-targeting vaccine outcome. Hum. Vaccine Immunother..

[122] Gustafson C.E., Kim C., Weyand C.M., Goronzy J.J. (2020). Influence of immune aging on vaccine responses. J. Allergy Clin. Immunol..

[123] Keck S., Schmaler M., Ganter S., Wyss L., Oberle S., Huseby E.S., Zehn D., King C.G. (2014). Antigen affinity and antigen dose exert distinct influences on CD4 T-cell differentiation. Proc. Natl. Acad. Sci. USA.

[124] Fazilleau N., McHeyzer-Williams L.J., Rosen H., McHeyzer-Williams M.G. (2009). The function of follicular helper T cells is regulated by the strength of T cell antigen receptor binding. Nat. Immunol..

[125] Ise W., Inoue T., McLachlan J.B., Kometani K., Kubo M., Okada T., Kurosaki T. (2014). Memory B cells contribute to rapid Bcl6 expression by memory follicular helper T cells. Proc. Natl. Acad. Sci. USA.

[126] Warfel J.M., Edwards K.M. (2015). Pertussis vaccines and the challenge of inducing durable immunity. Curr. Opin. Immunol..

[127] Auladell M., Jia X., Hensen L., Chua B., Fox A., Nguyen T.H.O., Doherty P.C., Kedzierska K. (2019). Recalling the Future: Immunological Memory Toward Unpredictable Influenza Viruses. Front. Immunol..

[128] Huisman W., Martina B.E., Rimmelzwaan G.F., Gruters R.A., Osterhaus A.D. (2009). Vaccine-induced enhancement of viral infections. Vaccine.

[129] Su S., Du L., Jiang S. (2020). Learning from the past: Development of safe and effective COVID-19 vaccines. Nat. Rev. Microbiol..

[130] Walker J.C., Smolders M.A., Gemen E.F., Antonius T.A., Leuvenink J., de Vries E. (2011). Development of lymphocyte subpopulations in preterm infants. Scand. J. Immunol..

[131] Kollmann T.R., Kampmann B., Mazmanian S.K., Marchant A., Levy O. (2017). Protecting the Newborn and Young Infant from Infectious Diseases: Lessons from Immune Ontogeny. Immunity.

[132] Vono M., Eberhardt C.S., Auderset F., Mastelic-Gavillet B., Lemeille S., Christensen D., Andersen P., Lambert P.H., Siegrist C.A. (2019). Maternal Antibodies Inhibit Neonatal and Infant Responses to Vaccination by Shaping the Early-Life B Cell Repertoire within Germinal Centers. Cell Rep..

[133] Ygberg S., Nilsson A. (2012). The developing immune system—From foetus to toddler. Acta Paediatr..

[134] Zandvoort A., Timens W. (2002). The dual function of the splenic marginal zone: Essential for initiation of anti-TI-2 responses but also vital in the general first-line defense against blood-borne antigens. Clin. Exp. Immunol..

[135] Siegrist C.A., Aspinall R. (2009). B-cell responses to vaccination at the extremes of age. Nat. Rev. Immunol..

[136] Mastelic B., Kamath A.T., Fontannaz P., Tougne C., Rochat A.F., Belnoue E., Combescure C., Auderset F., Lambert P.H., Tacchini-Cottier F. (2012). Environmental and T cell-intrinsic factors limit the expansion of neonatal follicular T helper cells but may be circumvented by specific adjuvants. J. Immunol..

[137] Morbach H., Eichhorn E.M., Liese J.G., Girschick H.J. (2010). Reference values for B cell subpopulations from infancy to adulthood. Clin. Exp. Immunol..

[138] Adkins B., Leclerc C., Marshall-Clarke S. (2004). Neonatal adaptive immunity comes of age. Nat. Rev. Immunol..

[139] Huygens A., Lecomte S., Tackoen M., Olislagers V., Delmarcelle Y., Burny W., Van Rysselberge M., Liesnard C., Larsen M., Appay V. (2015). Functional Exhaustion Limits CD4+ and CD8+ T-Cell Responses to Congenital Cytomegalovirus Infection. J. Infect. Dis..

[140] Risdon G., Gaddy J., Horie M., Broxmeyer H.E. (1995). Alloantigen priming induces a state of unresponsiveness in human umbilical cord blood T cells. Proc. Natl. Acad. Sci. USA.

[141] Morris M.C., Surendran N. (2016). Neonatal Vaccination: Challenges and Intervention Strategies. Neonatology.

[142] Burt T.D. (2013). Fetal regulatory T cells and peripheral immune tolerance in utero: Implications for development and disease. Am. J. Reprod. Immunol..

[143] Boer M.C., Joosten S.A., Ottenhoff T.H. (2015). Regulatory T-Cells at the Interface between Human Host and Pathogens in Infectious Diseases and Vaccination. Front. Immunol..

[144] Sautois B., Fillet G., Beguin Y. (1997). Comparative cytokine production by in vitro stimulated mononucleated cells from cord blood and adult blood. Exp. Hematol.

[145] Saso A., Kampmann B. (2017). Vaccine responses in newborns. Semin. Immunopathol..

[146] Wood N., Siegrist C.A. (2011). Neonatal immunization: Where do we stand?. Curr. Opin. Infect. Dis..

[147] Heldwein K.A., Liang M.D., Andresen T.K., Thomas K.E., Marty A.M., Cuesta N., Vogel S.N., Fenton M.J. (2003). TLR2 and TLR4 serve distinct roles in the host immune response against Mycobacterium bovis BCG. J. Leukoc. Biol..

[148] Marchant A., Goetghebuer T., Ota M.O., Wolfe I., Ceesay S.J., De Groote D., Corrah T., Bennett S., Wheeler J., Huygen K. (1999). Newborns develop a Th1-type immune response to Mycobacterium bovis bacillus Calmette-Guerin vaccination. J. Immunol..

[149] Rousseau M.C., El-Zein M., Conus F., Legault L., Parent M.E. (2016). Bacillus Calmette-Guerin (BCG) Vaccination in Infancy and Risk of Childhood Diabetes. Paediatr. Perinat. Epidemiol..

[150] Thostesen L.M., Kjaergaard J., Pihl G.T., Birk N.M., Nissen T.N., Aaby P., Jensen A.K.G., Olesen A.W., Stensballe L.G., Jeppesen D.L. (2018). Neonatal BCG vaccination and atopic dermatitis before 13 months of age: A randomized clinical trial. Allergy.

[151] Morra M.E., Kien N.D., Elmaraezy A., Abdelaziz O.A.M., Elsayed A.L., Halhouli O., Montasr A.M., Vu T.L., Ho C., Foly A.S. (2017). Early vaccination protects against childhood leukemia: A systematic review and meta-analysis. Sci. Rep..

[152] Demirjian A., Levy O. (2009). Safety and efficacy of neonatal vaccination. Eur. J. Immunol..

[153] Vojtek I., Dieussaert I., Doherty T.M., Franck V., Hanssens L., Miller J., Bekkat-Berkani R., Kandeil W., Prado-Cohrs D., Vyse A. (2018). Maternal immunization: Where are we now and how to move forward?. Ann. Med..

[154] Klein Klouwenberg P., Bont L. (2008). Neonatal and infantile immune responses to encapsulated bacteria and conjugate vaccines. Clin. Dev. Immunol..

[155] Landers C.D., Chelvarajan R.L., Bondada S. (2005). The role of B cells and accessory cells in the neonatal response to TI-2 antigens. Immunol. Res..

[156] Frasca D., Blomberg B.B. (2016). Inflammaging decreases adaptive and innate immune responses in mice and humans. Biogerontology.

[157] Fuentes E., Fuentes M., Alarcon M., Palomo I. (2017). Immune System Dysfunction in the Elderly. An. Acad. Bras. Cienc..

[158] Pera A., Campos C., Lopez N., Hassouneh F., Alonso C., Tarazona R., Solana R. (2015). Immunosenescence: Implications for response to infection and vaccination in older people. Maturitas.

[159] Nikolich-Zugich J. (2018). The twilight of immunity: Emerging concepts in aging of the immune system. Nat. Immunol..

[160] Gibson K.L., Wu Y.C., Barnett Y., Duggan O., Vaughan R., Kondeatis E., Nilsson B.O., Wikby A., Kipling D., Dunn-Walters D.K. (2009). B-cell diversity decreases in old age and is correlated with poor health status. Aging Cell.

[161] Ciabattini A., Nardini C., Santoro F., Garagnani P., Franceschi C., Medaglini D. (2018). Vaccination in the elderly: The challenge of immune changes with aging. Semin. Immunol..

[162] Frasca D., Riley R.L., Blomberg B.B. (2007). Aging murine B cells have decreased class switch induced by anti-CD40 or BAFF. Exp. Gerontol..

[163] Pritz T., Lair J., Ban M., Keller M., Weinberger B., Krismer M., Grubeck-Loebenstein B. (2015). Plasma cell numbers decrease in bone marrow of old patients. Eur. J. Immunol..

[164] Howard W.A., Gibson K.L., Dunn-Walters D.K. (2006). Antibody quality in old age. Rejuvenation Res..

[165] Frasca D., Landin A.M., Lechner S.C., Ryan J.G., Schwartz R., Riley R.L., Blomberg B.B. (2008). Aging down-regulates the transcription factor E2A, activation-induced cytidine deaminase, and Ig class switch in human B cells. J. Immunol..

[166] Blomberg B.B., Frasca D. (2013). Age effects on mouse and human B cells. Immunol. Res..

[167] Frasca D., Van der Put E., Riley R.L., Blomberg B.B. (2004). Reduced Ig class switch in aged mice correlates with decreased E47 and activation-induced cytidine deaminase. J. Immunol..

[168] Colonna-Romano G., Bulati M., Aquino A., Pellicano M., Vitello S., Lio D., Candore G., Caruso C. (2009). A double-negative (IgD-CD27-) B cell population is increased in the peripheral blood of elderly people. Mech. Ageing Dev..

[169] Han S., Yang K., Ozen Z., Peng W., Marinova E., Kelsoe G., Zheng B. (2003). Enhanced differentiation of splenic plasma cells but diminished long-lived high-affinity bone marrow plasma cells in aged mice. J. Immunol..

[170] den Braber I., Mugwagwa T., Vrisekoop N., Westera L., Mogling R., de Boer A.B., Willems N., Schrijver E.H., Spierenburg G., Gaiser K. (2012). Maintenance of peripheral naive T cells is sustained by thymus output in mice but not humans. Immunity.

[171] Malek T.R., Bayer A.L. (2004). Tolerance, not immunity, crucially depends on IL-2. Nat. Rev. Immunol..

[172] Ahmed M., Lanzer K.G., Yager E.J., Adams P.S., Johnson L.L., Blackman M.A. (2009). Clonal expansions and loss of receptor diversity in the naive CD8 T cell repertoire of aged mice. J. Immunol..

[173] Goronzy J.J., Fang F., Cavanagh M.M., Qi Q., Weyand C.M. (2015). Naive T cell maintenance and function in human aging. J. Immunol..

[174] Renkema K.R., Li G., Wu A., Smithey M.J., Nikolich-Zugich J. (2014). Two separate defects affecting true naive or virtual memory T cell precursors combine to reduce naive T cell responses with aging. J. Immunol..

[175] Sylwester A.W., Mitchell B.L., Edgar J.B., Taormina C., Pelte C., Ruchti F., Sleath P.R., Grabstein K.H., Hosken N.A., Kern F. (2005). Broadly targeted human cytomegalovirus-specific CD4+ and CD8+ T cells dominate the memory compartments of exposed subjects. J. Exp. Med..

[176] Virgin H.W., Wherry E.J., Ahmed R. (2009). Redefining chronic viral infection. Cell.

[177] Naylor K., Li G., Vallejo A.N., Lee W.W., Koetz K., Bryl E., Witkowski J., Fulbright J., Weyand C.M., Goronzy J.J. (2005). The influence of age on T cell generation and TCR diversity. J. Immunol..

[178] Bektas A., Schurman S.H., Sen R., Ferrucci L. (2017). Human T cell immunosenescence and inflammation in aging. J. Leukoc. Biol..

[179] Goodwin K., Viboud C., Simonsen L. (2006). Antibody response to influenza vaccination in the elderly: A quantitative review. Vaccine.

[180] Young B., Zhao X., Cook A.R., Parry C.M., Wilder-Smith A., MC I.C. (2017). Do antibody responses to the influenza vaccine persist year-round in the elderly? A systematic review and meta-analysis. Vaccine.

[181] Lefebvre J.S., Masters A.R., Hopkins J.W., Haynes L. (2016). Age-related impairment of humoral response to influenza is associated with changes in antigen specific T follicular helper cell responses. Sci. Rep..

[182] Jain S., Self W.H., Wunderink R.G., Fakhran S., Balk R., Bramley A.M., Reed C., Grijalva C.G., Anderson E.J., Courtney D.M. (2015). Community-Acquired Pneumonia Requiring Hospitalization among U.S. Adults. N. Engl. J. Med..

[183] Schmader K.E., Johnson G.R., Saddier P., Ciarleglio M., Wang W.W., Zhang J.H., Chan I.S., Yeh S.S., Levin M.J., Harbecke R.M. (2010). Effect of a zoster vaccine on herpes zoster-related interference with functional status and health-related quality-of-life measures in older adults. J. Am. Geriatr. Soc..

[184] Tseng H.F., Harpaz R., Luo Y., Hales C.M., Sy L.S., Tartof S.Y., Bialek S., Hechter R.C., Jacobsen S.J. (2016). Declining Effectiveness of Herpes Zoster Vaccine in Adults Aged ≥60 Years. J. Infect. Dis..

